# Monitoring monthly surface water dynamics of Dongting Lake using Sentinel-1 data at 10 m

**DOI:** 10.7717/peerj.4992

**Published:** 2018-06-20

**Authors:** Liwei Xing, Xinming Tang, Huabin Wang, Wenfeng Fan, Guanghui Wang

**Affiliations:** 1 College of Resource Environment and Tourism, Capital Normal University, Beijing, China; 2 Satellite Surveying and Mapping Application Center, National Administration of Surveying, Mapping and Geoinformation, Beijing, China

**Keywords:** Dongting Lake, Sentinel-1, Surface water areas, Threshold-based classification, Monthly dynamic changes

## Abstract

High temporal resolution water distribution maps are essential for surface water monitoring because surface water exhibits significant inner-annual variation. Therefore, high-frequency remote sensing data are needed for surface water mapping. Dongting Lake, the second-largest freshwater lake in China, is famous for the seasonal fluctuations of its inundation extents in the middle reaches of the Yangtze River. It is also greatly affected by the Three Gorges Project. In this study, we used Sentinel-1 data to generate surface water maps of Dongting Lake at 10 m resolution. First, we generated the Sentinel-1 time series backscattering coefficient for VH and VV polarizations at 10 m resolution by using a monthly composition method. Second, we generated the thresholds for mapping surface water at 10 m resolution with monthly frequencies using Sentinel-1 data. Then, we derived the monthly surface water distribution product of Dongting Lake in 2016, and finally, we analyzed the inner-annual surface water dynamics. The results showed that: (1) The thresholds were −21.56 and −15.82 dB for the backscattering coefficients for VH and VV, respectively, and the overall accuracy and Kappa coefficients were above 95.50% and 0.90, respectively, for the VH backscattering coefficient, and above 94.50% and 0.88, respectively, for the VV backscattering coefficient. The VV backscattering coefficient achieved lower accuracy due to the effect of the wind causing roughness on the surface of the water. (2) The maximum and minimum areas of surface water were 2040.33 km^2^ in July, and 738.89 km^2^ in December. The surface water area of Dongting Lake varied most significantly in April and August. The permanent water acreage in 2016 was 556.35 km^2^, accounting for 19.65% of the total area of Dongting Lake, and the acreage of seasonal water was 1525.21 km^2^. This study proposed a method to automatically generate monthly surface water at 10 m resolution, which may contribute to monitoring surface water in a timely manner.

## Introduction

The global extent of 304 million lakes (4.2 million km^2^ in area) cover about 3% of the earth’s surface (Downing et al., 2006; Pekel et al., 2016). These lakes provide water supply, flood control, irrigation functions, support for human survival, social development, and ecosystem stability (Barrow, 2016; Song et al., 2016). However, climate change and human activities may strongly affect lake acreage and result in an inner-annual surface water distribution variation that affects the climate, biological diversity, and human wellbeing (Cheng et al., 2016; Chiang, Perng & Liou, 2017; Klein et al., 2017). Therefore, timely monitoring of surface water could satisfy a need for ecological and environmental construction, lake resources management, flood prediction, and drought resistance.

Remote sensing imagery observes the land surface in a timely and accurate way, and has been widely used to monitor the dynamics of surface water (Feng et al., 2012; Giardino et al., 2013; Hereher, 2015; Kutser et al., 2005; Sun et al., 2014; Veettil et al., 2016; Wright & Pilger, 2008). Several global land cover products have mapped the surface water distributions, such as GLobCover (Arino et al., 2008), MOD44W (Carroll et al., 2009), GLOBCOVER2009 (Bontemps et al., 2011), GLCNMO2 (Tateishi et al., 2014), and GlobalLand 30 (Chen et al., 2015), but the global land cover products were produced at a low temporal frequency, which cannot adequately describe the inner-annual surface water change. Low-resolution data with high temporal resolution, such as AVHRR and MODIS data, has also been used to map the inner-annual body of water changes (Feng et al., 2012; Jain et al., 2006; Kang & Hong, 2016; Klein et al., 2017; Sun et al., 2014), but the coarse resolution of these images led to misclassification due to the problem of mixed pixels, while images with higher spatial resolution, such as those from Sentinel-2, Landsat and China’s HJ satellites (Feng et al., 2016; Liao et al., 2014; Verpoorter et al., 2014), always have low temporal frequency and irregular image time series because of clouds. For example, the whole of Dongting Lake was covered by cloud-free Landsat 8 images only in July 2016. We have generated monthly composited Sentinel-2 image time series in 2016, but only images in May, July, August, and December have low cloud coverage. So proposing a water surface identification method which is not affected by the cloud is of importance for inner-annual water surface dynamics monitoring (Pekel et al., 2016), particularly in regions with high cloud cover.

Synthetic aperture radar (SAR) data have been considered a promising tool to monitor water dynamics because they are cloud-proof and illumination-independent (Gstaiger et al., 2012; Long, Fatoyinbo & Policelli, 2014). However, SAR data have been less often used, because some high spatial resolution SAR data, such as TerraSAR-X (X-band), COSMO-SkyMed (X-band), and RADARSAT-2 (C-band) (Gstaiger et al., 2012; Pulvirenti et al., 2011), are always expensive, which limits the use of the data at large scale. Freely available data, such as ENVISAT ASAR, have a moderate resolution of 150 m, which cannot accurately describe the interannual dynamics of the surface water (Kuenzer et al., 2013; Matgen et al., 2011). With the recent launch of the Sentinel-1 satellite and the free access to its products (Torres et al., 2012), SAR data have both high temporal and spatial resolution, which can be used to extract surface water extent and assess its dynamics more efficiently (Cazals et al., 2016). In addition, radar altimeter satellites are cloud-proof and can also be used to monitor surface water area (Duan & Bastiaanssen, 2013; Sima & Tajrishy, 2013).

Dongting Lake acts as a great retention basin for the Yangtze River (Ding & Li, 2011). Surface water at Dongting Lake presents inner-annual variation because of the dynamics of water levels in the Yangtze River and the adjustment of the Three Gorges Project (Li et al., 2011). Some studies have focused on monitoring the surface water variation at Dongting Lake using time series data (Ding & Li, 2011; Hu et al., 2015), but this research used MODIS or ENVISAT ASAR data at 150, 250, and 500 m or lower spatial resolution, which cannot accurately monitor the seasonal variation of surface water area. Thus, there is a need to monitor the inner-annual dynamics of Dongting Lake at higher spatial resolution, such as using Sentinel images with 10 m resolution. Sentinel-1 data have high spatiotemporal resolution, are free of cost, and resistant to cloud cover, which makes this a suitable alternative. There have been studies on extracting surface water area using Sentinel-1 data (Čotar, Oštir & Kokalj, 2016; Pham-Duc, Prigent & Aires, 2017), but the drawback of these studies is that the methods are complicated. Therefore, the main objectives of this study are (1) to create a SAR time series using Sentinel-1 data with a monthly composition strategy for 2016; (2) to find the universal threshold for monthly water extraction; (3) to monitor the monthly surface water area of Dongting Lake in 2016 based on the threshold; and (4) to analyze the monthly dynamic change in the surface water area of Dongting Lake.

## Study Area and Datasets

### Study area

**Figure 1:**
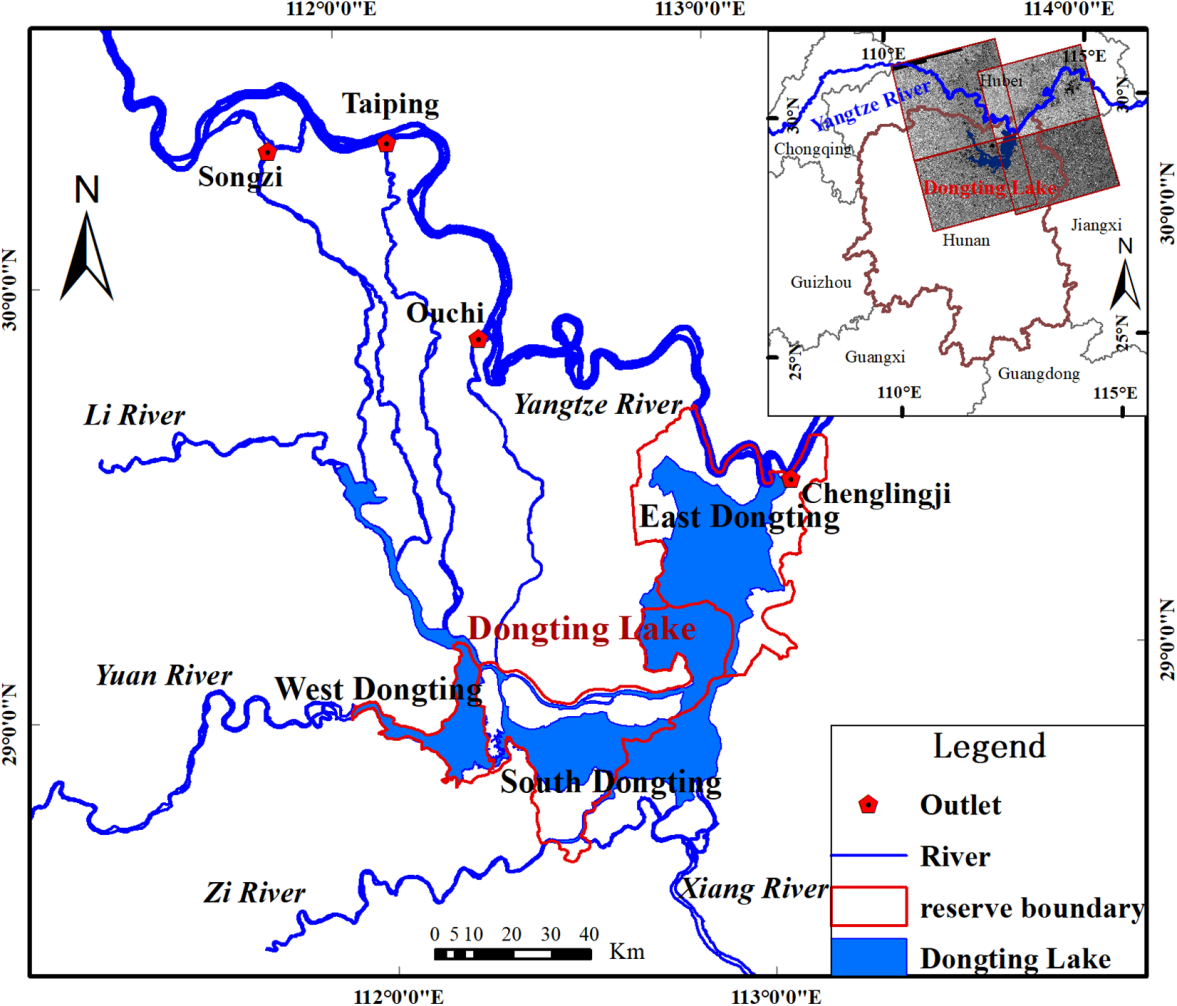
Location of study area.

Dongting Lake (28°30′ to 30°20′N, 110°40′∼113°10′E) is the second-largest freshwater lake in China, located at the middle reaches of the Yangtze River Basin, with an extensive catchment area (Fig. 1) (Ding & Li, 2011). The lake has the Xiangjiang River, the Zi River, the Yuan River, and the Lishui River as its south and west tributaries; the Miluo River and the Xinqiang River as its east tributaries; the Taiping estuary and the Ouchi estuary as the bleeders of Yangtze River water flow, and the lake water discharged back into the Yangtze River at the northeast outlet near Chenglingji (Li et al., 2013; Zhang et al., 2006). In addition, Dongting Lake is a typical passing reservoir, which adjusts both the inflow from and outflow to the mid-reaches of the Yangtze River. During flood season between April and September, the lake provides storage for river flood waters. During the dry season between October and March, it provides water to the river to allow river transportation to continue without significant interruption (Zhang et al., 2006). Because of its important geographic location, Dongting Lake includes three national wetland nature reserves: the East Dongting wetland, the South Dongting wetland, and the West Dongting wetland, which were included in the List of Ramsar Sites in 1992 and 2002 (http://www.ramsar.org/wetland/china). It also supports shipping and fisheries, provides irrigation water, and regulates the local climate. Therefore, monitoring the short term dynamic change in the surface water area of Dongting Lake has direct and significant effects on preventing floods, the stability of the wetland ecosystem and its biodiversity.

### Datasets

#### Remote sensing imagery

Sentinel-1 includes two radar satellites in one constellation (Sentinel-1A and -1B) which were launched by the European Space Agency (ESA) on April 3, 2014 and April 25, 2016, respectively (Plank, 2014). Sentinel satellites provide C-Band (center frequency 5.405 GHz) images in both singular and dual polarization. The repeat cycle of a single satellite is 12 days, whereas a six-day repeat pass observation can be achieved with the two satellites (Nagler et al., 2016). Four acquisition modes: strip map, interferometric wide swath, extra wide swath, and wave) can be acquired for different processing levels (Cazals et al., 2016). For this study, we used Level-1 ground range detected images, which belong to the IW mode with dual polarization (VV/VH) (Cazals et al., 2016), produced by Sentinel-1A (https://scihub.copernicus.eu/dhus/#/home) in 2016 over Dongting Lake. Covering the whole of Dongting Lake requires mosaicing four image scenes taken at approximately the same time. The images used in this study are listed in Table 1. The data product has a swath width of 250 km at a resolution of 5 × 20 m in the range and azimuth directions, respectively, and an imagery pixel spacing of 10 m in ground geometry (Torres et al., 2012).

**Table 1:** The dates of Sentinel-1 images taken at Dongting Lake.

Year	Month	Day
2016	January	17th, 24th
	February	10th, 17th
	March	5th, 12th, 29th
	April	5th, 22nd, 29th
	May	16th, 23rd
	June	9th
	July	3rd, 22nd
	August	3rd, 20th, 27th
	September	25th
	October	2nd, 7th, 14th, 19th, 26th, 31st
	November	7th, 12th, 19th, 24th
	December	1st, 6th, 13th, 18th, 25th, 30th

#### Training and validation samples

In this study, training and validation samples were collected to determine the threshold and assess accuracy for each of the 12 months. The training samples were obtained using a stratified random sampling method for January, April, July, and October. More specifically, the minimum enclosing rectangle of the Dongting Lake boundary was divided into nine columns and nine rows resulting in 81 cells, and at least 10 sites with a center of 100 × 100 m pure pixels were randomly sampled from each cell. The training samples for each month were classified as water or non-water using images from Google Earth (http://earth.google.com), Landsat ETM+, and Landsat OLI (https://earthexplorer.usgs.gov/) in the same month. The land cover types in the non-water training samples included buildings, forest, shrub, rice field, and marsh. The number of training samples for these four months is shown in Table 2.

**Table 2:** The number of training samples for each month.

Month	Water	Non-water
January	141	669
April	173	637
July	203	607
October	133	677

Initially, 1,000 validation points that were interpreted in the same way as the training samples were randomly created using ArcGIS 10.3, and only the points with unambiguous interpretation were kept. For each month, 200 water validation samples and 400 non-water validation samples were used to assess the classification accuracy.

## Methodology

**Figure 2:**
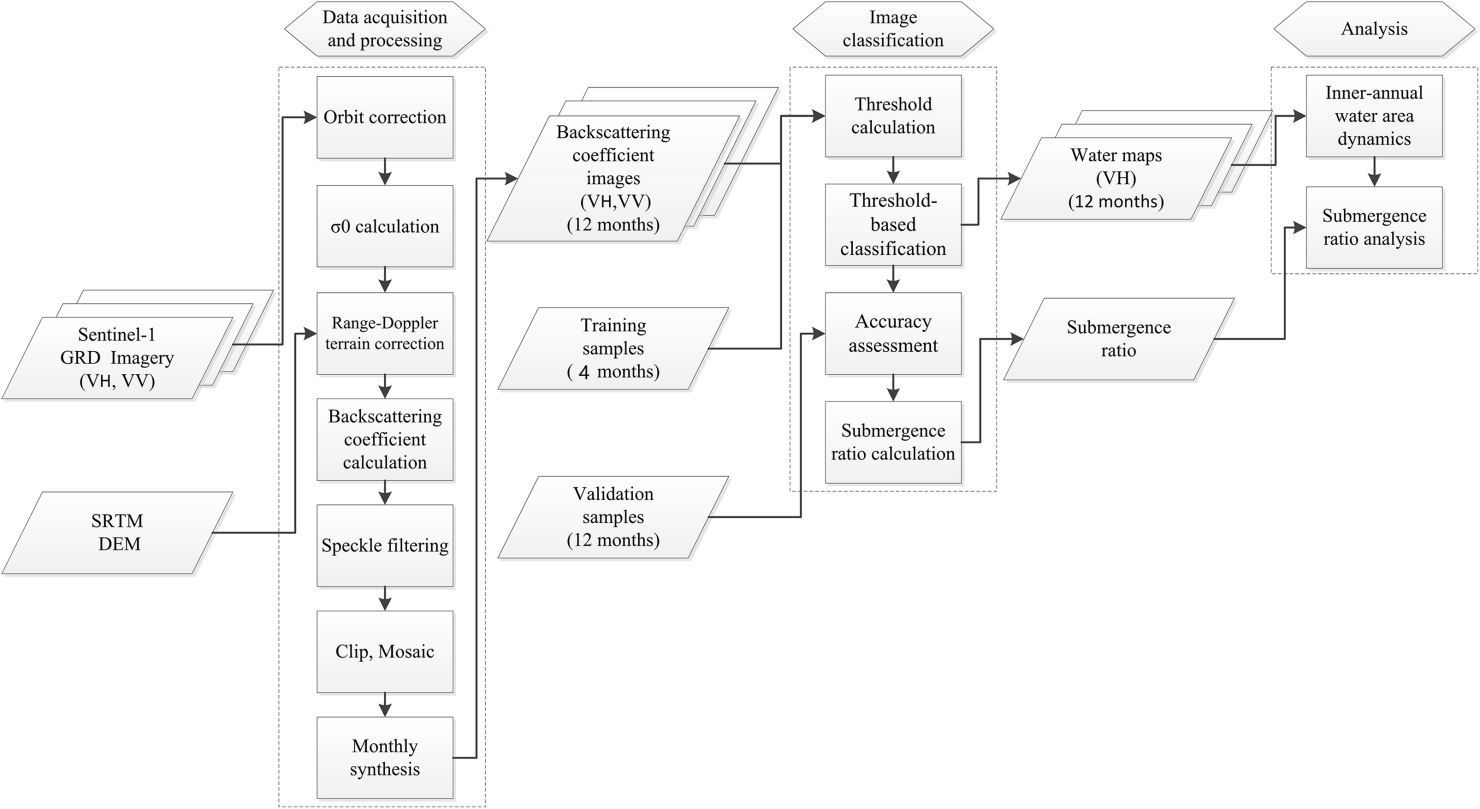
Flowchart of the methodology followed in the study.

Figure 2 shows the overall methodology used in this study. First, sentinel application platform software was used to preprocess Sentinel-1A imagery to acquire the backscattering coefficients for VH and VV polarizations. We then generated the time series of backscattering coefficients using the monthly synthesis method. Second, the thresholds for the backscattering coefficients for VH and VV polarizations were calculated based on the training samples. The thresholds were then used to extract the surface water at Dongting Lake for each month based on the backscattering coefficient for VH and VV polarizations. After assessing the classification accuracy, the dynamic change of surface water area at Dongting Lake was analyzed.

### Data preprocessing

When the radar electromagnetic wave is specularly reflected by calm surface waters, water pixels present lower backscattering than in bare soil or vegetation pixels. So, when the backscattering coefficient is the lowest, the soil moisture content is highest. In our study, the backscattering coefficient for VH and VV polarizations were used to extract the surface water area of Dongting Lake.

Sentinel application platform software (https://sentinel.esa.int/web/sentinel/toolboxes/sentinel-1) provided by the ESA was utilized to preprocess the Sentinel-1A imagery. The workflow included six steps: (1) Sentinel-1A images were allocated to orbit files. (2) σ_0_ bands were created from Sentinel-1A images through radiometric calibration. (3) The Range Doppler Terrain Correction was used to orthorectify σ_0_ bands with SRTM 3 s as digital elevation model. (4) The backscattering coefficients (in dB) was acquired from the orthorectified σ_0_ band by the equation 10 × log10 (σ_0_) (Li, Shao & Zhang, 2011). (5) Speckle noise was removed by using a single product speckle filter method with a window size of 5 × 5 pixels (Gupta & Gupta, 2007; Schmidt & Skidmore, 2004; Tian et al., 2015). (6) The median value of all pixels at the same location for each month was used to extract the monthly surface water extent.

### Threshold-based classification

During the past decade, many methods for water mapping with SAR images have been developed, such as visual interpretation (Brivio et al., 2002; MacIntosh & Profeti, 1995), image texture analysis (Schumann et al., 2005), histogram thresholding (Matgen et al., 2004), the edge detection approach (Schumann, Di Baldassarre & Bates, 2009), and image statistic-based active contour models (Horritt, Mason & Luckman, 2001; Schumann et al., 2005). In some studies, the thresholding technique has been viewed as an efficient and simple water mapping method (Schumann, Di Baldassarre & Bates, 2009). In general, the key point is determining the threshold (Bazi, Bruzzone & Melgani, 2007). Empirical methods have been most commonly used for determining thresholds (Gstaiger et al., 2012). Empirically defined gray-level thresholds for classification depend much on the operator’s ability to distinguish different gray-scale tones and their knowledge of water body image characteristics, which may lead to variable accuracy due to the subjective evaluation. Other methods for selecting the threshold include the Otsu method (Otsu, 1979), the maximum entropy method (Kapur, Sahoo & Wong, 1985) and the minimum interclass variance method (Radhika, Sekhar & Venkatesha, 2009), all of which are computationally complex and time-consuming. In this study, we used a simple and efficient method to select the threshold.

**Figure 3:**
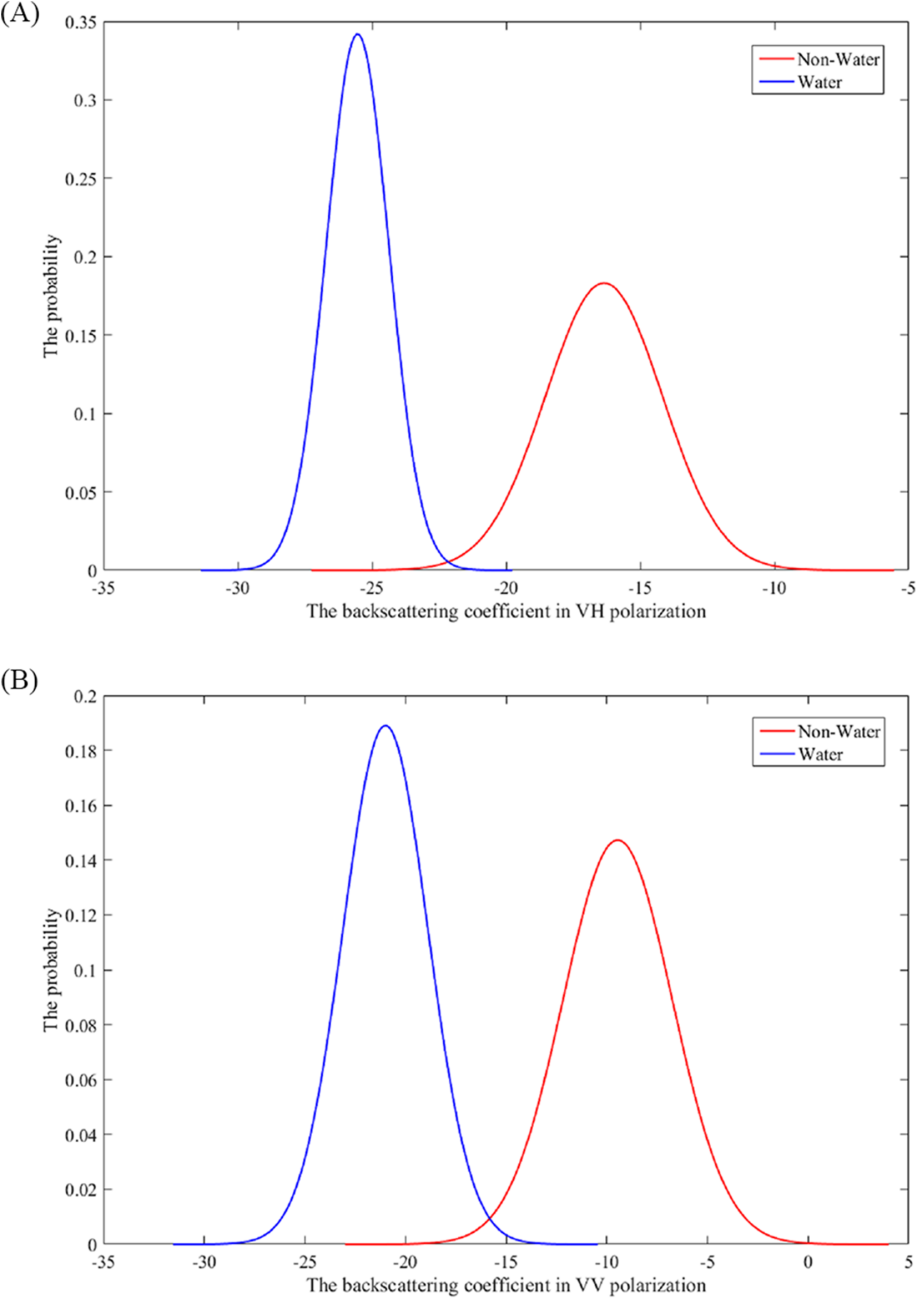
The normally distributed probability density curve of the backscattering coefficient for VH or VV polarization (A and B) for water and non-water.

The method was based on the normal probability density function of the training sample of water and non-water. The Shapiro–Wilk test in SPSS release 25.0 was performed on four sets of water and non-water training samples. Normality was determined by checking the backscattering coefficient for VH or VV polarizations. For all four sets of training samples data, all tests have a *p*-value 0.05, which indicates a normal distribution of data. The plots of the normal distribution probability density functions of the water and non-water training samples presented two obvious single peaks (Fig. 3). The water and non-water each corresponded to a peak (Fig. 3). There was a trough between the two peaks. The probabilities of the water and non-water were both lower in the trough region. The value in the trough could be considered as the threshold that separates water and non-water. Thus, we generated the normal probability density function using the training samples, and then used the *x*-axis value of the intersection of the two probability density function plots as the threshold for identifying surface water. Meanwhile, as water and non-water have the same probability at this threshold, the accuracy of both the water and non-water results is guaranteed to be at a maximum. In addition, this method can also prevent the case where the accuracy of one class is ensured while the accuracy of others is ignored.

### Accuracy evaluation

Accuracy assessment of the surface water distribution map is an important part in the image classification procedure. In this study, overall accuracy (OA) (Eq. (1)), user’s accuracy (UA) (Eq. (2)), producer’s accuracy (PA) (Eq. (3)), and Kappa coefficient (Eq. (4)) were used to evaluate the classification accuracy of Sentinel-1A (Congalton, 1991). The series of accuracy metrics can be derived from a confusion matrix which is the most frequently used method in accuracy evaluation (Foody, 2002):

(1) $${p_{\rm{o}}} = \mathop \sum \limits_{i = 1}^r {{{p_{ii}}} \over N}$$

(2) $${p_{{u_i}}} = {{{p_{ii}}} \over {{p_{i + }}}}$$

(3) $${p_{{{\rm{A}}_i}}} = {{{p_{ii}}} \over {{p_{ + i}}}}$$

(4) $${\rm{Kappa}} = {{N*\mathop \sum \nolimits_{i = 1}^r {p_{ii}}-\mathop \sum \nolimits_{i = 1}^r \left({{p_{i + }}*{P_{ + i}}} \right)} \over {{N^2}-\mathop \sum \nolimits_{i = 1}^r \left({{p_{i + }}*{p_{ + i}}} \right)}}$$

where *p*_o_ is the OA; ${p_{{u_i}}}$ is the UA; ${p_{{{\rm{A}}_i}}}$ is the PA; *p_ii_* is the number of correctly classified samples of class *i*; *r* is the number of classes; *N* is the number of training samples; *p_i+_* is the number of classified samples of class *i*; and *p_+i_* is the number of training samples of class *i*.

### Dynamic change analysis method

To understand the water dynamics change characteristics and influences at Dongting Lake, the submergence ratio was defined as the probability of a pixel being inundated by water for one year (Eq. (5)):

(5) $${P_{{\rm{ratio}}}} = {n \over N}*100\ {\text %} $$

where *P*_ratio_ represents the submergence ratio; *n* is the inundation number of each pixel observed by Sentinel-1A in a year; and *N* is the observation times of Sentinel-1A (equal to 12 in this study).

## Results and Discussion

### Classification accuracy

Validation samples were used to access the surface water mapping accuracies. The results (Table 3) showed that both VH and VV backscattering coefficients achieved high mapping accuracies, and the water and non-water types were well-distinguished based on the threshold-based classification method. The OA and kappa coefficients of all classification results were above 94.50% and 0.88, respectively. The producer’s and user’s accuracies for both water and non-water types were above 91.50% and 91.67%. It seems that the errors in the results were mainly omissions or commissions of water pixels around the edges of the lakes. To prove the reliability of results, training samples were obtained for another eight months and a support vector machine (SVM) was used to map the water of Dongting Lake. The classification accuracies based on SVM demonstrated that the OA and kappa coefficients of all classification results were above 95.00% and 0.89 (Table 4). The producer’s and user’s accuracies for both water and non-water types were above 92.00% and 92.65% (Table 4). The results indicate that threshold-based classification accuracies were close to the accuracies based on SVM (only 2.24% lower). Thus, the classification errors cannot due to using of a simple algorithm. In contrast, the threshold-based classification method was universal and effective.

**Table 3:** Monthly “Water” and “Non-Water” identification accuracy based on threshold method.

	VH				VV			
Month	OA (%)	Kappa	Water	Non-Water	OA (%)	Kappa	Water	Non-Water
			PA/UA (%)	PA/UA (%)			PA/UA (%)	PA/UA (%)
January	96.67	0.93	95.50/94.55	97.25/97.74	95.83	0.91	94.00/93.53	96.75/96.99
February	96.33	0.92	95.00/94.06	97.00/97.49	96.20	0.91	93.66/95.05	97.50/96.77
March	95.50	0.90	93.50/93.03	96.50/96.74	94.50	0.88	91.50/91.96	96.00/95.76
April	96.33	0.92	95.50/93.63	96.75/97.73	95.67	0.90	94.00/93.07	96.50/96.98
May	97.00	0.93	96.00/95.05	97.50/97.99	96.15	0.91	94.79/93.90	96.85/97.32
June	95.50	0.90	94.00/92.61	96.25/96.98	95.00	0.89	93.50/91.67	95.75/96.72
July	96.67	0.93	96.50/93.69	96.75/98.22	95.50	0.90	94.00/92.61	96.25/96.98
August	97.33	0.94	97.00/95.10	97.50/98.48	96.67	0.93	95.50/94.55	97.25/97.74
September	96.83	0.93	95.00/95.48	97.75/97.51	96.00	0.91	94.00/94.00	97.00/97.00
October	97.17	0.94	96.00/95.52	97.75/97.99	95.17	0.89	93.50/92.12	96.00/96.73
November	96.30	0.92	94.79/94.34	97.07/97.31	96.10	0.91	93.75/94.66	97.30/96.83
December	96.33	0.92	95.00/94.06	97.00/97.49	95.50	0.90	94.00/92.61	96.25/96.98

**Note:**

OA, overall accuracy; UA, user accuracy; PA, producer accuracy.

**Table 4:** Monthly “Water” and “Non-Water” identification accuracy based on SVM method.

	VH				VV			
Month	OA (%)	Kappa	Water	Non-Water	OA (%)	Kappa	Water	Non-Water
			PA/UA (%)	PA/UA (%)			PA/UA (%)	PA/UA (%)
January	97.50	0.94	97.00/95.57	97.75/98.49	96.17	0.91	93.50/94.92	97.50/96.77
February	97.00	0.93	96.00/95.05	97.50/97.99	96.83	0.93	94.50/95.94	98.00/97.27
March	96.00	0.91	94.50/93.56	96.75/97.24	95.00	0.89	92.00/95.94	96.50/96.02
April	96.33	0.92	94.50/94.50	97.25/97.25	95.67	0.90	93.50/93.50	96.75/96.75
May	97.67	0.95	97.00/96.04	98.00/98.49	96.83	0.93	95.50/95.02	97.50/97.74
June	96.17	0.91	95.00/93.60	96.75/97.48	95.67	0.90	94.50/92.65	96.25/97.22
July	97.33	0.94	97.50/94.66	97.25/98.73	96.17	0.91	94.50/94.03	97.00/97.24
August	97.50	0.94	97.00/95.57	97.75/98.49	97.00	0.93	95.00/95.96	98.00/97.51
September	97.00	0.93	95.00/95.96	98.00/97.51	95.90	0.91	93.50/93.97	97.07/96.84
October	98.17	0.96	97.00/97.49	98.75/98.50	95.83	0.91	94.50/93.10	96.50/97.23
November	97.00	0.93	96.00/95.05	97.50/97.99	96.17	0.91	94.50/94.03	97.00/97.24
December	96.83	0.93	96.00/94.58	97.25/97.98	95.67	0.90	94.00/93.07	96.50/96.98

**Note:**

OA, overall accuracy; UA, user accuracy; PA, producer accuracy.

By comparison, the results further indicated the superiority and higher performance of the VH backscattering coefficient as compared with the VV backscattering coefficient. Accordingly, the classification results based on the VH backscattering coefficient were used to monitor the monthly changes and the submergence ratio of Dongting Lake in 2016.

MODIS data have high temporal resolution, the revisit time is one day. It is widely used in monitoring the dynamics of surface water area. However, due to the problem of mixed pixels, the accuracies of these researches are lower. The map producer and map user accuracies of surface water from MODIS are always below 90%, which means that part of the surface water area is not detected by MODIS (Li et al., 2009). It may be that other advanced methods can improve the quantity accuracy; however, MODIS data cannot identify details of the surface water and small water bodies precisely at this time. The advantage of our research is that data used in this study exhibit a high spatial resolution, which can reduce misclassification, so the accuracy of our study is higher than MODIS and the results of classification are more reliable. Landsat data have 30 m of spatial resolution. The classification accuracies of water surface maps based on Landsat data are always higher than 90% (Zhou et al., 2017). However, Landsat data cannot provide monthly cloud-free images, especially in regions with high precipitation, thus such images cannot be used for monitoring inner-annual water dynamics.

### Water identification threshold

**Figure 4:**
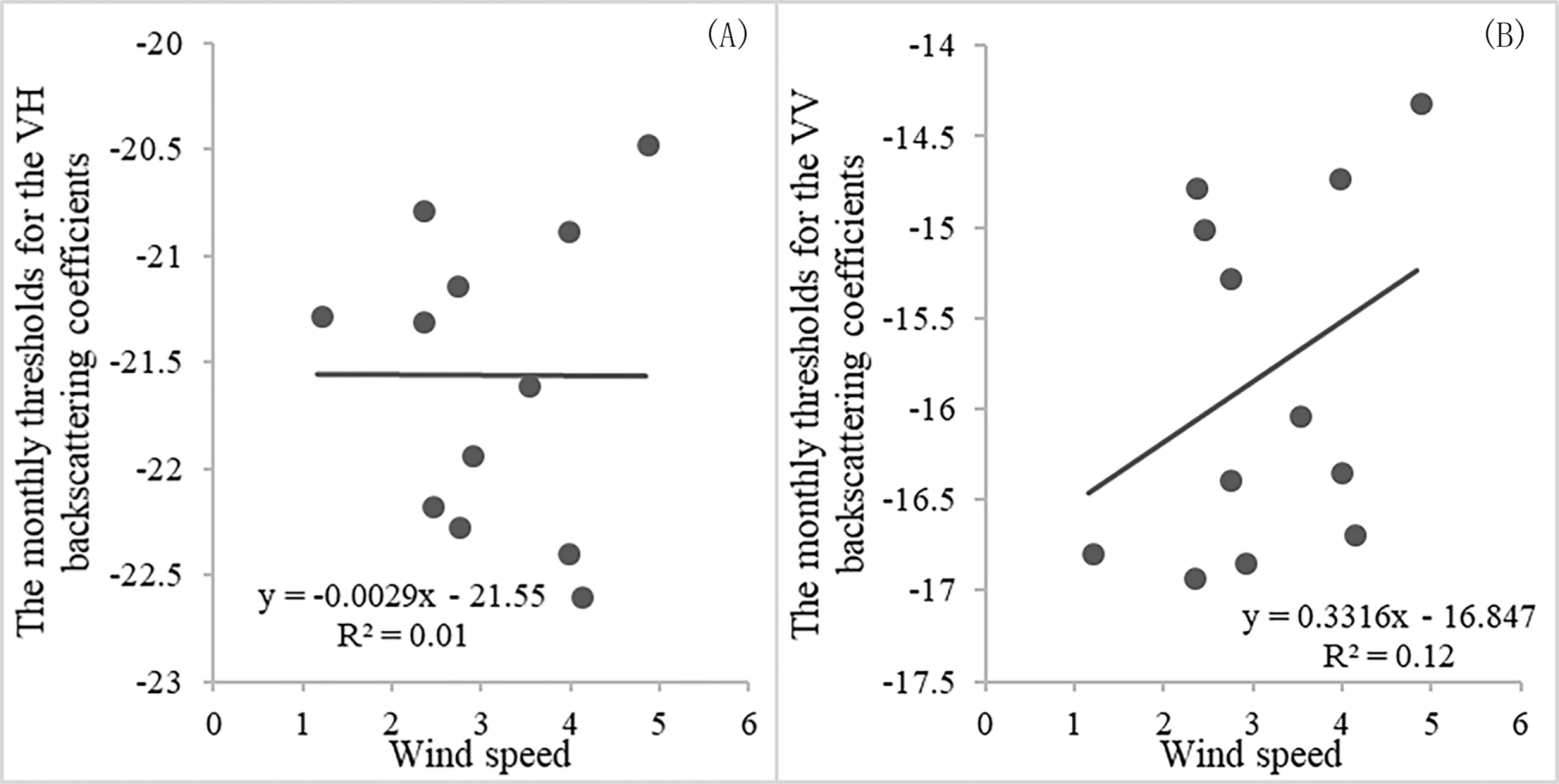
The relationship between wind speed and threshold for VH or VV backscattering coefficient (A and B). Note: wind speed is the square root of v wind speed square plus u wind speed square.

The thresholds for the VH and VV backscattering coefficients were −21.56 and −15.82 dB. Zeng et al. (2017) selected the value −16.35 dB as a threshold for the VV backscattering coefficient to extract the surface water area of Poyang Lake using Sentinel-1 SAR imagery. Their thresholds for surface water identification were similar to the thresholds used in our research. We also obtained monthly VH and VV backscattering coefficient thresholds using the method proposed in this paper and corresponding monthly training samples. The results are shown in Table 5. The monthly thresholds for VH backscattering coefficient were between −22.59 and −20.46 dB. The monthly thresholds for VV backscattering coefficient were between −16.91 and −14.30 dB. The thresholds either for VH or VV backscattering coefficient varied slightly over time. The thresholds in this paper for the VH and VV backscattering coefficients were −21.56 and −15.82 dB, which was close to the monthly thresholds. The key factor influencing the threshold is the status of water surface. Wind is an important factor that disturbs the water surface. Monthly longitudinal (u) and lateral (v) wind speeds data at 10 m above the water surface of Dongting Lake were acquired from the ERA-Interim data (http://app.ecmwf.int/datasets/). The v and u wind speeds were between 0.47 m/s and 4.58 m/s. Dongting Lake experiences gentle breeze with grade-3 wind-forces throughout the year. Wind speeds vary slightly during the year. Figure 4 is an illustration of a fitting curve for monthly wind speeds and thresholds for VH or VV backscattering coefficient. The two fitted linear functions (*R*^2^ = 0.01 and *R*^2^ = 0.12) demonstrated that the threshold was not obviously related to the wind speed. Therefore, seasonal changes of the effect of wind speed on water surface over the year can be ignored. In addition, the accuracy assessment showed that all the results reached a very high accuracy (91.50–98.48%), which indicated that thresholds in this study were feasible.

**Table 5:** Monthly thresholds for VH or VV backscattering coefficient and monthly v and u wind speed at 10 m above the water surface of Dongting Lake.

	VH (dB)	VV (dB)	v-wind (m/s)	u-wind (m/s)
January	−22.16	−14.99	2.19	1.04
February	−22.26	−16.37	2.66	0.55
March	−21.92	−16.83	1.50	2.45
April	−21.60	−16.02	3.02	1.76
may	−22.38	−16.33	3.59	1.65
June	−22.59	−16.67	3.98	0.99
July	−20.46	−14.30	4.58	1.57
August	−20.77	−14.77	1.74	1.54
September	−21.30	−16.91	2.26	0.51
October	−21.27	−16.78	1.07	0.47
November	−20.87	−14.71	3.79	1.11
December	−21.13	−15.26	2.19	1.59

By comparison, the accuracies of water and non-water classification based on the VH backscattering coefficient were slightly higher than that based on the VV backscattering coefficient. This finding confirmed that the VH polarization was more sensitive to the presence of surface water than the VV polarization, which was consistent with the conclusion found by Pham-Duc, Prigent & Aires (2017). In addition, wind-induced surface roughness over open water can influence the backscattering coefficients, especially for the VV polarization. The average annual breeze speed at Dongting Lake was with wind-force of three grades (Liu et al., 2009). The wind may affect VV backscatter coefficients more and further lead to more misclassifications between water and non-water than VH-polarized data. In addition, the water surface identification method solely uses Sentinel-1 data, which is not affected by clouds. This method is therefore valuable for the water surface monitoring work in the region affected by high cloud coverage and precipitation.

### Inner-annual water area dynamics

**Figure 5:**
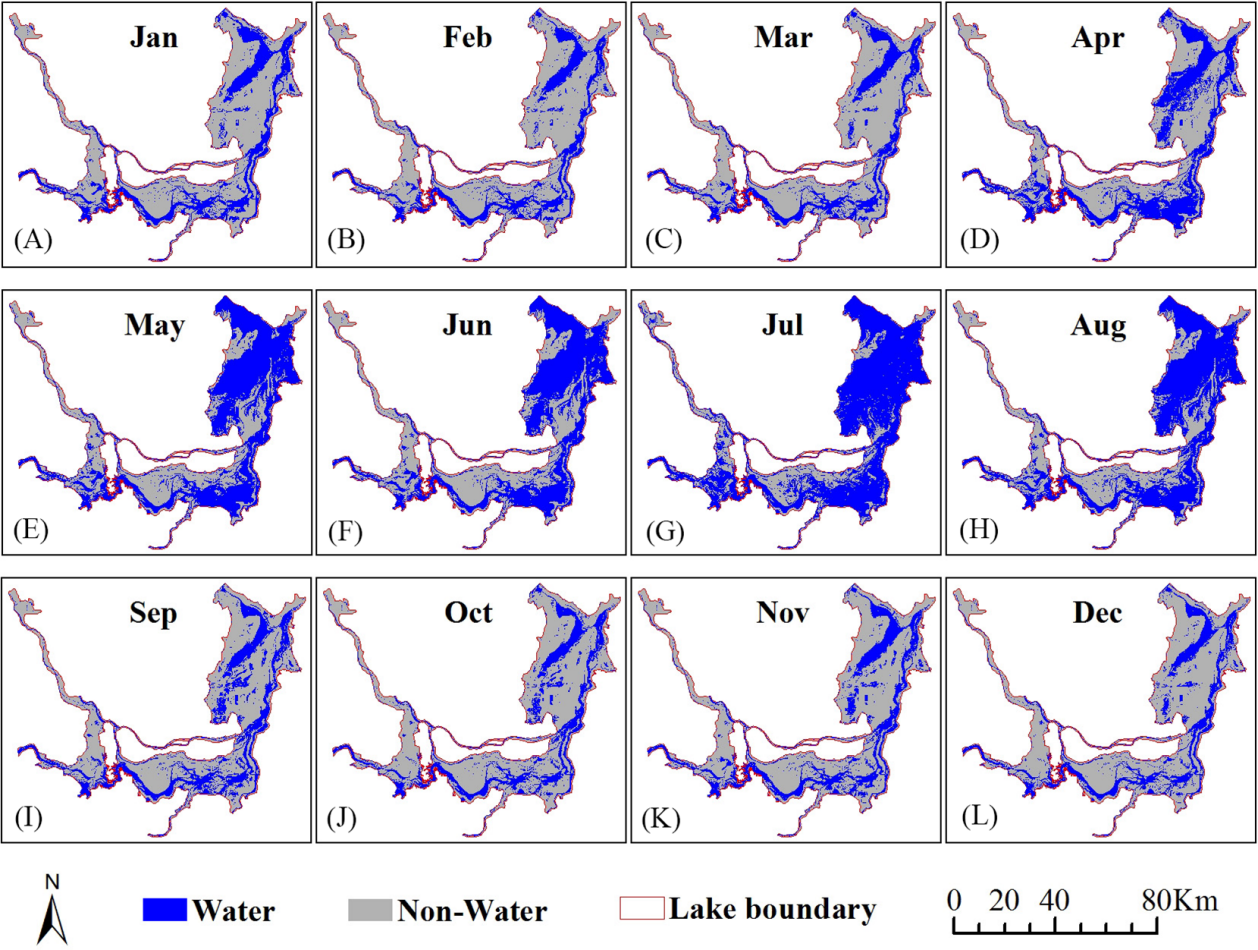
Spatiotemporal distribution of surface water at Dongting Lake in 2016 based on the VH backscattering coefficient (A–L).

The classification results were obtained using the universal threshold to distinguish water and non-water from the VH backscattering coefficient images. Figure 5 shows the monthly surface water acreage of Dongting Lake in 2016. Surface water distributions showed significant seasonal variation. During the rainy season, the lake was in a vast expanse of water, but in the dry season, the lake lost water. The surface water acreage of Dongting Lake changed from 738.89 km^2^ in December to 2040.33 km^2^ in July. During the rainy season (April to August), the lake area was larger than 1,000 km^2^, with an average of 1572.59 km^2^. During the dry season (September to March), the lake area was less than 887.38 km^2^, with an average of 809.75 km^2^. The annual surface water area of Dongting Lake extracted from MODIS images ranged from 440 to 1,900 km^2^ from 2005 to 2009 (Huang, Li & Xu, 2012). The difference between the minimum values is greater than about 300 km^2^. The main reason is that the spatial resolution of MODIS is too coarse to allow extraction of the small water bodies, or to identify the water detail in certain regions of Dongting Lake that are narrow, especially during the dry season. In addition, the change characteristics of the water area at Dongting Lake showed obvious regional differences. The seasonal change of water area happened mainly in the East Dongting wetland and the South Dongting wetland. The spatial extent of the water area of the West Dongting wetland remained relatively stable.

**Figure 6:**
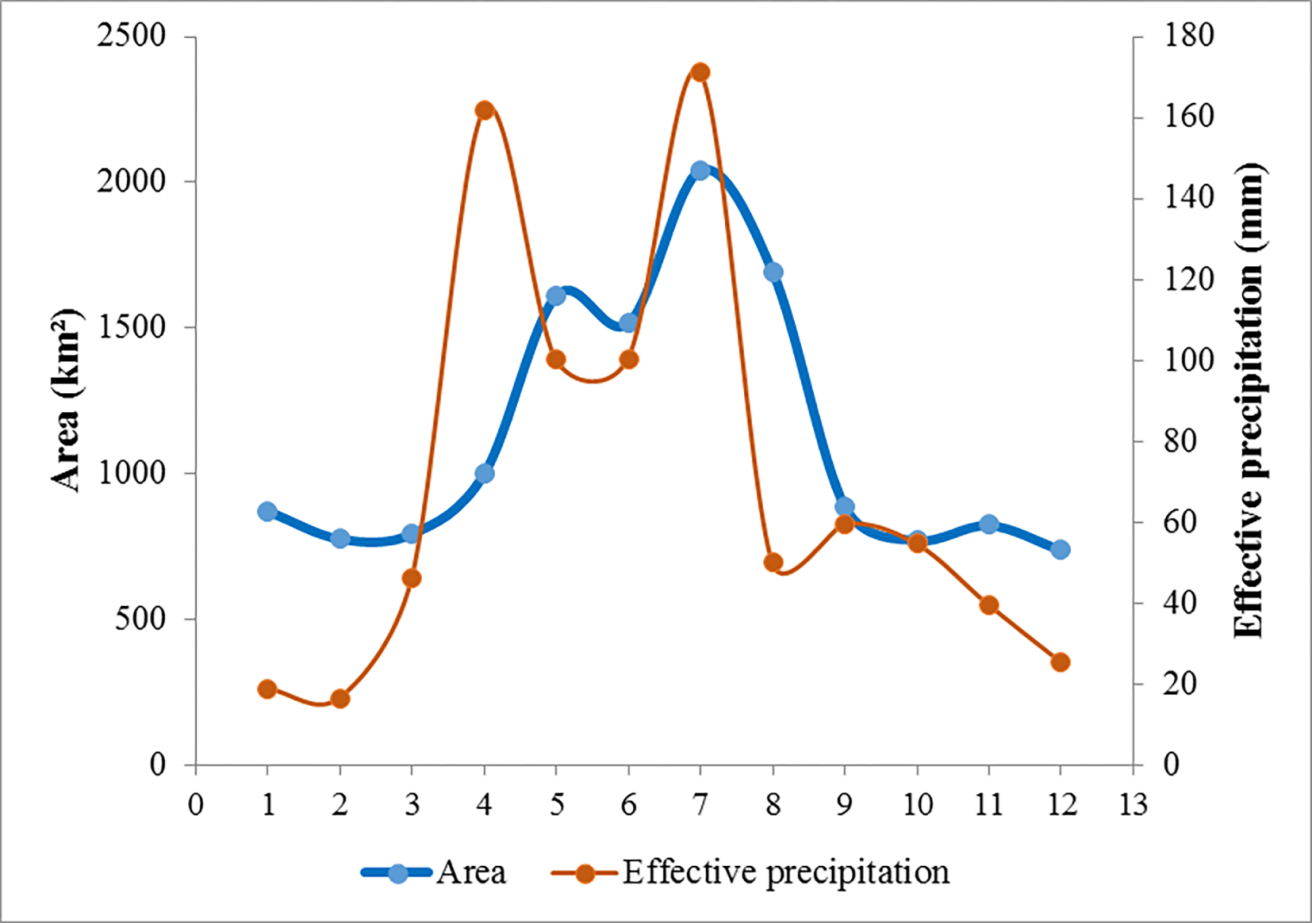
Changes in the average monthly effective precipitation and surface water area of Dongting Lake obtained on the VH backscattering coefficient.

In 2016, the water area of Dongting Lake began to increase in April and reached a peak in July, then started to decline in September and reached a low in December (Fig. 6). The maximum loss in surface area was 803.23 km^2^ with a variation rate of 47.51% from August to September. The maximum increase in area was 609.67 km^2^ with a variation rate of 60.81% from April to May. The average changed acreage was 268.50 km^2^. The variation rate in each monthly interval showed that there were eight changes greater than 10%. The average variation rate was 21.38%.

The precipitation, evaporation, runoff, and infiltration were all major influences on Dongting Lake’s surface water areas. However, only monthly precipitation and evaporation data for 2016 were acquired from the ERA-Interim data, which were downloaded from the European Centre for Medium-Range Weather Forecasts (http://app.ecmwf.int/datasets/). In our study, effective monthly precipitation equals the monthly precipitation minus the monthly evaporation. Figure 6 shows the correlation between the surface water area of Dongting Lake and the effective precipitation over time. The tendencies of both are consistent. The surface water area of Dongting Lake increased as the effective precipitation increased, and deceased as the effective precipitation decreased. Both the surface water area and the effective precipitation reached a maximum value in July. Therefore, the effective precipitation was one of the most important factors that influenced the dynamic changes of surface water area of Dongting Lake.

### Submergence ratio analysis

**Figure 7:**
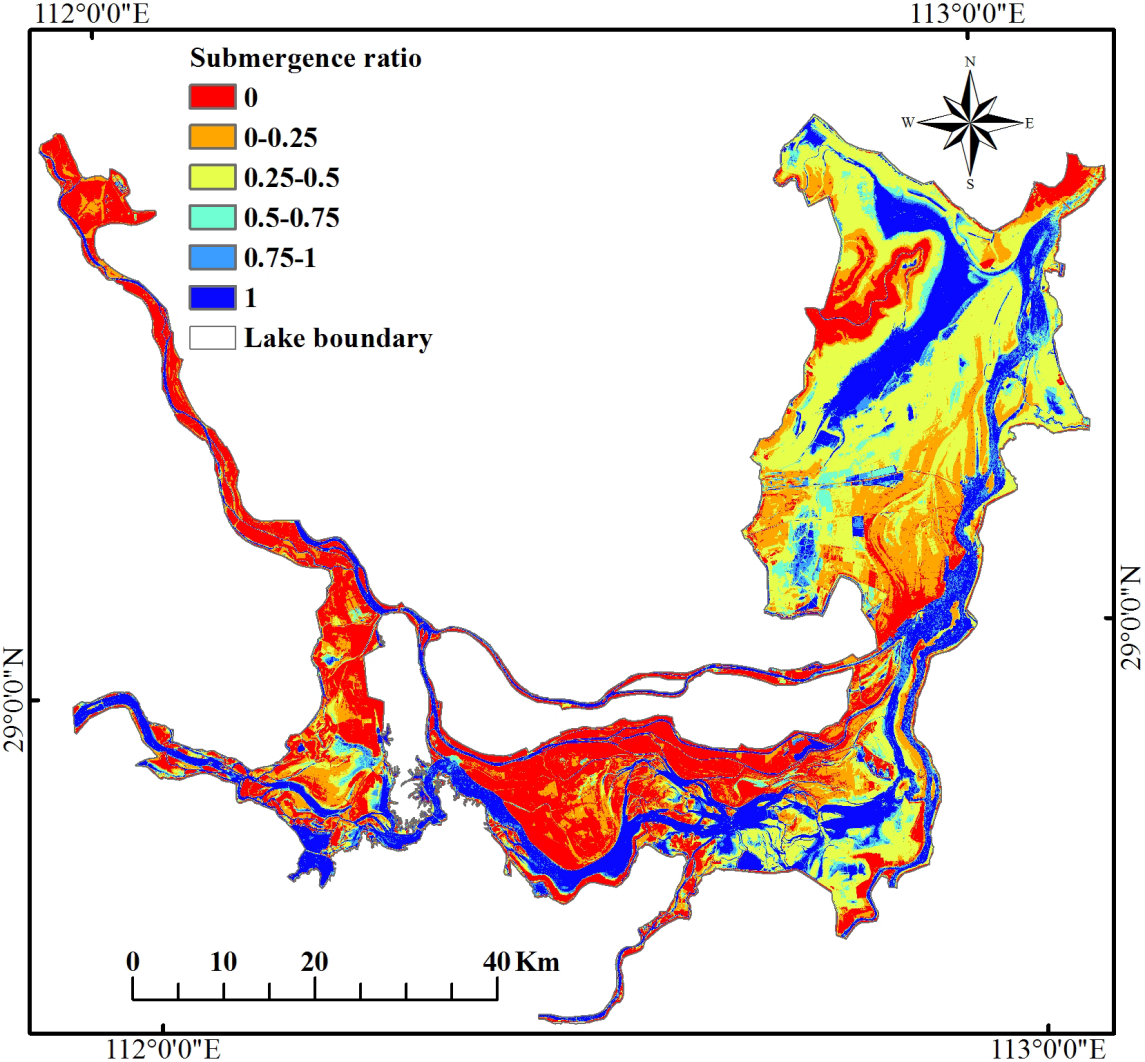
Submergence ratios at Dongting Lake from January 1, 2016 to December 31, 2016.

**Figure 8:**
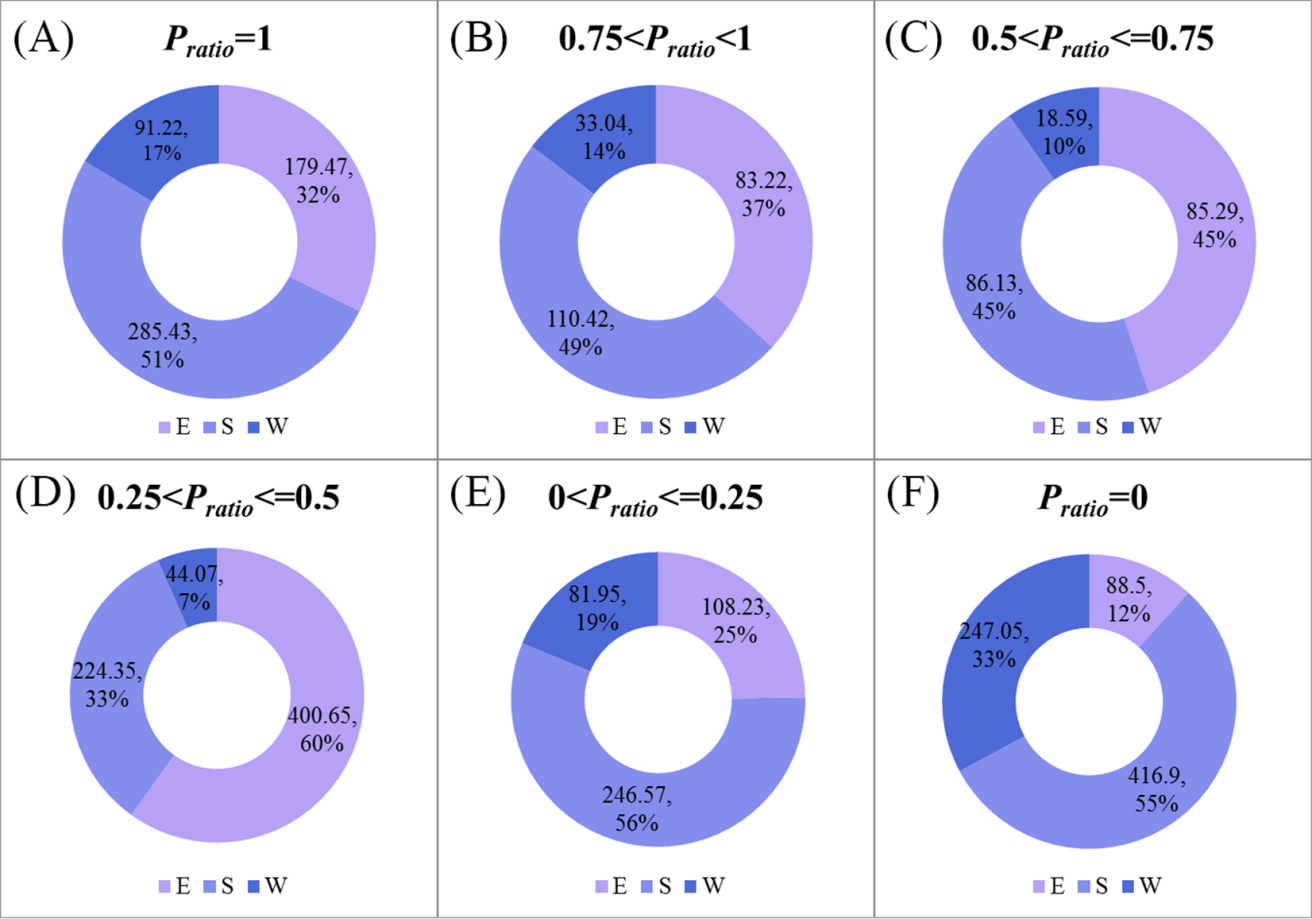
The area (km^2^) and proportion of three national wetland nature reserves at Dongting Lake in the six submergence ratios levels (A–F). Note: *P*_ratio_ represents the submergence ratios; E represents the East Dongting wetland; S represents the South Dongting wetland; W represents the West Dongting wetland.

The submergence ratio shows the spatiotemporal dynamics change of surface water area at Dongting Lake in 2016 (Fig. 7). The overall change characteristics of the value of submergence ratio presented a decrease from the lake center to the edges. The acreage of the permanent water that was submerged the entire year in 2016 (submergence ratio = 1) was 556.35 km^2^, accounting for 19.65% of the total area of Dongting Lake. The permanent water was mainly distributed in the central district of Dongting Lake. Approximately 83% of permanent water was located in the south Dongting Lake (51%) and the East Dongting Lake (32%) (Fig. 8). The area of seasonal water was 1525.21 km^2^, accounting for 53.87% of the total area of Dongting Lake, and these regions should receive more attention due to their higher risk of flooding. The seasonal water was generally distributed around permanent water. The seasonal water (0.75 < submergence ratio < 1, and 0 < submergence ratio ≤ 0.25) were all mainly located in the South Dongting Lake (49%, 56%). The seasonal water (0.25 < submergence ratio ≤ 0.50) was mainly located in the East Dongting Lake (60%). The acreage of seasonal water (0.50 < submergence ratio ≤ 0.75) was equal in both the East Dongting Lake (45%) and the South Dongting Lake (45%). Regardless of whether the water was permanent, water or seasonal water were less than 20% in the West Dongting Lake. In this study area, there were still 749.93 km^2^ of surface area that was not submerged at any time during the year (defined as non-water land, with a submergence ratio of 0), and 88% of the non-water land was located in the East Dongting Lake and the South Dongting Lake.

**Figure 9:**
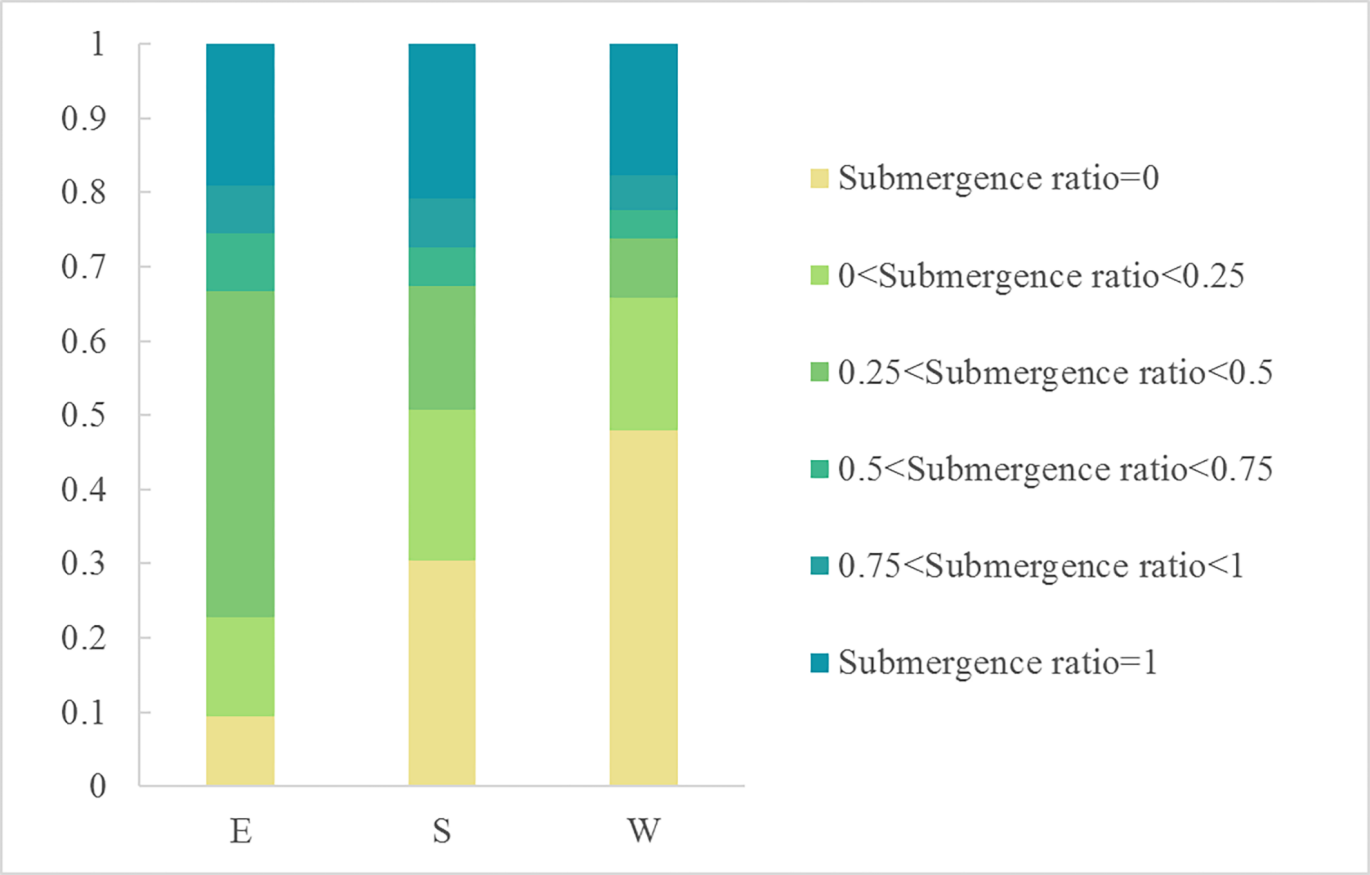
The proportion of the six submergence ratio levels in three national wetland nature reserves of Dongting. Note: E represents the East Dongting wetland; S represents the South Dongting wetland; W represents the West Dongting wetland.

As a whole, the permanent surface water (submergence ratio = 1) of the East Dongting Lake, the South Dongting Lake, and the West Dongting Lake accounted for 20% of the subset lake area. Meanwhile, there were still some differences in the spatial characteristics of these three subset lakes. In the West Dongting wetland, the non-water (submergence ratio = 0) accounted for the largest proportion of the total surface area (48%). However, the permanent water accounted for the largest surface area in both the East Dongting Lake (72%) and the South Dongting Lake (49%) (Fig. 9).

## Conclusion

Most existing studies about the surface water area of Dongting Lake use images at 150, 250, and 500 m or lower spatial resolution. In this study, we found thresholds to extract the monthly surface water of Dongting Lake based on Sentinel-1 data at 10 m spatial resolution, and monitored the inner-annual dynamics of Dongting Lake in 2016. The main conclusions are as follows.

1. The thresholds for identifying the surface water using VH and VV were −21.56 and −15.82 dB for VH and VV, respectively; overall classification accuracies and kappa coefficients were all above 94.50% and 0.88. Producer’s and user’s accuracies of the water and non-water classifications for each month were all above 91.50% and 91.67%, respectively. Notably, the thresholds are universal and effective.
2. VH obtained higher accuracy than VV when identifying surface water. The OA, PA, UA, and Kappa coefficients for each month calculated using backscattering coefficients from VH were all above 95.50%, 93.50%, 93.03%, and 0.90, respectively. The OA, PA, UA, and Kappa coefficients for each month calculated using backscattering coefficients from VV were all above 94.50%, 91.50%, 91.96%, and 0.88, respectively. The main reason was that the surface water of Dongting Lake was not calm through the year, due to the effect of the wind causing roughness on the surface of the water.
3. The surface water acreage of Dongting Lake in 2016 began to increase in April, and then began to decrease in August. The surface water acreage was the largest in July, at 2040.33 km^2^, and the smallest in December, at 738.89 km^2^.

This study used Sentinel-1 data at 10 m spatial resolution to extract the surface water area of Dongting Lake for each month in 2016. The temporal resolution of Sentinel-1 is 12 days. There are at least two images per month in the same place. Dongting Lake belongs to a tropical monsoon climate, which experiences a high frequency of cloudy and rainy weather throughout the year. Thus, it would not be possible to reach even a similar level using the free optical data at the approximate spatial resolution. The inadequacy of this method is that the vessels affect the water classification and were not eliminated in this research. This impact in the total area and monthly dynamic of Dongting Lake can be ignored because the area impacted by such vessels is small, compared to the total area of Dongting Lake. In addition, Sentinel-1 imagery is acquired systematically; it is possible to monitor the dynamic change of the surface water at Dongting Lake at a higher spatial and temporal resolution. In our study, we analyzed only the correlation between the monthly surface water area and the effective precipitation. As more meteorological data is obtained, we will continue to study the more detailed reasons for the dynamic change in surface water at the Dongting Lakes.

## Supplemental Information

10.7717/peerj.4992/supp-1 Supplemental Information 1 Validation samples in December 2016.

10.7717/peerj.4992/supp-2 Supplemental Information 2 Validation samples in March 2016.

10.7717/peerj.4992/supp-3 Supplemental Information 3 Validation samples in June 2016.

10.7717/peerj.4992/supp-4 Supplemental Information 4 Training samples in July 2016.

10.7717/peerj.4992/supp-5 Supplemental Information 5 Validation samples in August 2016.

10.7717/peerj.4992/supp-6 Supplemental Information 6 Validation samples in November 2016.

10.7717/peerj.4992/supp-7 Supplemental Information 7 Validation samples in February 2016.

10.7717/peerj.4992/supp-8 Supplemental Information 8 Validation samples in September 2016.

10.7717/peerj.4992/supp-9 Supplemental Information 9 Validation samples in April 2016.

10.7717/peerj.4992/supp-10 Supplemental Information 10 Training samples in April 2016.

10.7717/peerj.4992/supp-11 Supplemental Information 11 Training samples in October 2016.

10.7717/peerj.4992/supp-12 Supplemental Information 12 Validation samples in May 2016.

10.7717/peerj.4992/supp-13 Supplemental Information 13 Validation samples in July 2016.

10.7717/peerj.4992/supp-14 Supplemental Information 14 Validation samples in October 2016.

10.7717/peerj.4992/supp-15 Supplemental Information 15 Training samples in January 2016.

10.7717/peerj.4992/supp-16 Supplemental Information 16 Validation samples in January 2016.

## Additional Information and Declarations

### Data Availability

The following information was supplied regarding data availability: The training and validation samples data are provided as Supplemental files.
